# Comparative efficacy of non-pharmacological adjuvant therapies for quality of life in the patients with breast cancer receiving chemo- or radio-therapy: A protocol for systematic review and Bayesian network meta-analysis

**DOI:** 10.1097/MD.0000000000012096

**Published:** 2018-08-21

**Authors:** Zhiyun He, Ailin Song, Zhongtao Zhang, Youcheng Zhang, Xiaokang Liu, Lei Zhao, Xi Lv, Guosheng Ren, Yumin Li

**Affiliations:** a Colorectal Surgical Department of Lanzhou University Second Hospital; b VIP Surgical Department of Lanzhou University Second Hospital, Lanzhou; c Beijing Friendship Hospital of Capital Medical University; d National Digestive System Diseases Clinical Researching Center, Beijing; e General Surgery Department of Lanzhou University Second Hospital, Lanzhou; f The First Affiliated Hospital of Chongqing Medical University, Chongqing, China.

**Keywords:** breast cancer, chemotherapy, network meta-analysis, non-pharmacological, quality of life, radiotherapy

## Abstract

**Background:**

Breast cancer is the most frequently diagnosed cancer in women worldwide. When treated by chemotherapy and/or radiotherapy, there are various non-pharmacological adjuvant therapies (NPATs) recommended for helping the patients with breast cancer alleviate multiple side effects induced by chemotherapy and/or radiotherapy and improve quality of life (QoL). However, the existing evidence does not suggest the therapy with the best effectiveness among a variety of NPATs. This study is to compare the effectiveness of different NPATs on QoL in the patients with breast cancer using Bayesian network meta-analysis (NMA).

**Methods and analysis:**

We will conduct a comprehensive search strategy in the relevant databases (MEDLINE, EMBASE, Cochrane Central Register of Controlled Trials, Allied and Complementary Medicine Database, Cumulative Index to Nursing and Allied Health Literature, PsycINFO, World Health Organization (WHO), International Clinical Trials Registry Platform (ICTRP) search portal (http://apps.who.int/trialsearch/Default.aspx), Chinese Biomedical Literature Database, China National Knowledge Infrastructure, Wan Fang Data). The random or quasi-random controlled trails that compare different NPATs in patient with breast cancer who received the chemotherapy and/or radiotherapy will be included. We only focus on the outcome of QoL which can be assessed by a series of tools. The risk of bias for included studies will be appraised using the Cochrane Collaboration's tool for assessing risk of bias. The standard pairwise meta-analysis and a Bayesian NMA will be conducted.

**Ethics and dissemination:**

Ethical approval and patient consent are not required since this is an NMA based on published studies. We will submit our NMA to a peer-reviewed journal for publication.

**PROSPERO registration number:**

CRD42017078143.

## 1 Background

Breast cancer is one of the top frequently diagnosed cancers, with estimated 1,676,600 new cases and 521,900 deaths worldwide every year.^[1]^ For the patients with breast cancer, chemotherapy and/or radiotherapy play an essential role in reducing recurrence rate and improving overall survival rate, which has been presented in a range of existing researches.^[2–6]^ Moreover, the clinical guideline for breast cancer from National Comprehensive Cancer Network (NCCN) recommends numerous chemotherapy and radiotherapy regimens as well.^[7]^ However, there are various adverse effects (such as fatigue, nausea, vomiting, insomnia, anxiety, pain, phlebitis, peripheral neuropathy, alopecia, and mucositis) caused by chemotherapy and/or radiotherapy in patients with breast cancer^[8–11]^ which may reduce quality of life (QoL) and adherence of treatment.

Therefore, some types of adjuvant therapies, particularly non-pharmacological adjuvant therapies (NPATs) (such as relaxation, mindfulness-based stress reduction (MBSR), music therapy, massage;^[12]^ yoga, acupuncture, meditation, qigong, reflexology, and stress management^[13]^) are usually combined with chemotherapy and/or radiotherapy. In addition, evidence from the existing systematic reviews also finds that Tai chi and expressive writing have positive impact on QoL of patients with breast cancer.^[14,15]^ These NPATs do not cause any severe adverse side effects and are inexistent of drug interactions, which makes it more acceptable for patients compared with pharmacological adjuvant therapies.^[16]^ However, all of the existing meta-analyses only conducted pairwise meta-analysis to compare efficacy of different types of NPATs. In order to assess the effects of different types of NPATs on QoL of the patients with breast cancer, it is necessary to produce highly compelling and persuasive evidence to draw a firm conclusion.

Network meta-analysis (NMA)^[17]^ can be used for addressing this problem, which is able to evaluate the relative effectiveness among all the potential interventions and rank the order of interventions by estimated effect size as head-to-head comparisons are lacking. This study is a comprehensive systematic review and NMA on different types of NPATs for patients with breast cancer.

## 2 Objective

The objective of this study is to compare the effectiveness of different NPATs on QoL in the patients with breast cancer using Bayesian network meta-analysis of randomized clinical trials (RCTs).

## 3 Methods

### 3.1 Design

A systematic review and Bayesian NMA will be carried out in this study.

### 3.2 Registration

We registered the protocol of the present systematic review on the international prospective register of systematic reviews, PROSPERO (Register number: CRD42017078143). The protocol was conducted in accordant with the preferred reporting items for systematic review and meta-analysis protocol (PRISMA-P),^[18,19]^ and the PRISMA extension statement for reporting of systematic reviews incorporating NMAs of healthcare interventions.^[20]^

### 3.3 Eligibility criteria

#### 3.3.1 Types of studies

We plan to include truly random or quasi-random controlled trails. In addition, the relevant systematic reviews or meta-analyses will also be included to track their references.

#### 3.3.2 Type of patients

Adult women (age of eighteen or over) diagnosed as breast cancer and receiving chemotherapy and/or radiotherapy are eligible for this review, whereas patients will be excluded if metastasis is found in other organs.

#### 3.3.3 Type of interventions

The NPATs below will be included: yoga, acupuncture, meditation, qigong, reflexology, stress management, relaxation, music therapy, massage, acupressure, expressing writing, and Tai chi.

#### 3.3.4 Type of outcomes

We only focus on the outcome of QoL which can be assessed by a series of tools such as European Organization for Research and Treatment of Cancer (EORTC) Quality of Life Questionnaire (EORTC QLQ-C30), Functional Assessment of Cancer Therapy (FACT), McGill QoL Questionnaire, City of Hope QoL Questionnaire, Supportive Care Needs Survey (SCNS), 36-Item Short Form Health Survey (SF-36), Quality of Life in Adult Cancer Survivors (QLACS), etc.

### 3.4 Information source

The search strategy will be developed by consulting the librarians of Lanzhou University. And the information sources include the databases below:

MEDLINE (via PubMed), EMBASE, Cochrane Central Register of Controlled Trials, Allied and Complementary Medicine Database, Cumulative Index to Nursing and Allied Health Literature, PsycINFO, World Health Organization (WHO) International Clinical Trials Registry Platform (ICTRP) search portal (http://apps.who.int/trialsearch/Default.aspx), Chinese Biomedical Literature Database, China National Knowledge Infrastructure, Wan Fang Data

The references of included articles and reviews will be tracked to identify other relevant studies.

### 3.5 Search strategy

No limitation about language and publication date will be restricted. The search terms contain the relevant text words regarding breast cancer, chemotherapy, radiotherapy and quality of life. The details of PubMed search strategy are as follows:

#1 “Breast Neoplasms”[Mesh] OR “Breast Cancer Lymphedema”[Mesh] OR “Breast Neoplasm∗”[Title/Abstract] OR “Breast Tumor∗”[Title/Abstract] OR “Breast Carcinoma∗”[Title/Abstract] OR “Mammary Cancer∗”[Title/Abstract] OR “Mammary Carcinoma∗”[Title/Abstract] OR “Mammary Neoplasm∗”[Title/Abstract] OR “Mammary Tumor∗”[Title/Abstract]

#2 “Radiotherapy”[Mesh] OR “Drug Therapy”[Mesh] OR chemotherap∗[Title/Abstract] OR chemo-therap∗[Title/Abstract] OR “drug therapy”[Title/Abstract] OR irradiation[Title/Abstract] OR radiotherap∗[Title/Abstract] OR radio-therap∗[Title/Abstract] OR radiation[Title/Abstract] OR chemo-radio-therap∗[Title/Abstract] OR chemoradi∗[Title/Abstract] OR radiochemo∗[Title/Abstract]

#3 “Quality of Life”[Mesh] OR “Quality of Life”[Title/Abstract] OR “Life Quality”[Title/Abstract]

#4 #1 AND #2 AND #3

All the details of search strategy of the databases can be seen in the supplement file.

### 3.6 Study selection and data extraction

The results of electronic search will be imported to EndNote X7 literature management software for study selection which is composed of 2 steps: title and abstract selection, full-text selection. In the stage of title and abstract screening, the potentially relevant researches will be identified. And then full-texts will be reviewed to confirm eligible studies in the next step. The trials excluded and the reasons for their exclusion in the second stage will be listed and examined by a third reviewer.

We will conduct a standard data extraction form using Microsoft Excel 2013 to carry out data extraction. The following data will be collected: study characteristics (such as title, first author, publication type, publication year, country, journal, and the sponsor), study design (inclusion and exclusion criteria, generation of allocation sequence, allocation concealment, and blinding, length of follow-up), participant data (sample size, race, age, tumor stage, diagnostic criteria, time of diagnose, co-morbidities, and lost/withdrawal/abscission), details of chemotherapy or radiotherapy (dose, duration, and combination), details of interventions of interest (type, frequency, and duration), and outcomes (assessment tools, assessment time point, and assessment result).

A pilot test will be performed for literature selection and data extraction, and a “cheat sheet” with detailed definitions and examples will be developed to ensure high inter-rater reliability among the reviewers. Study selection and data extraction will be accomplished individually by 2 researchers. Any disagreements will be resolved by discussion, and conflicts will be solved by a third researcher.

### 3.7 Risk of bias assessment

The risk of bias of included RCTs will be appraised using the Cochrane Collaboration's tool for assessing risk of bias^[21]^ by 2 independent researchers, and conflicts will be resolved by a third researcher. The tool contains 7 domains, namely sequence generation, allocation concealment, blinding of participants and personnel, blinding of outcome assessment, incomplete outcome data, selective outcome reporting, and other sources of bias. Each domain is evaluated as low, high, and unclear risk of bias. We will record the explanation of the result of each domain.

### 3.8 Dealing with missing data

If the researchers didn’t report the important data (such as the standard deviation, standard errors of continue outcome), we will try to calculate it first using algebraic manipulation based on the reported information such as confidence interval (CI). If failed, we will contact the authors to obtain these data. If that would be still not possible, the methods suggested by Furukawa et al will be used to retrieve the missing data.^[22]^ The assumptions derived from these data will be tested through sensitivity analysis.

### 3.9 Standard pairwise meta-analysis

We will perform pairwise meta-analysis, using STATA V.12.0 software (Stata Corporation, College Station, Texas), and pool ORs with 95% CI for dichotomous outcomes and mean differences (MDs) with 95% CI for continue outcomes. We will assess heterogeneity of treatment effects across trials by c^2^ and I^2^ statistics. If the *P* value >.1 and *I*^2^ <50%, it means that there is no statistical heterogeneity, and the Mantel–Haenszel fixed effects model will be used for meta-analysis. If the *P* value <.1 and *I*^2^ >50%, subgroup analysis and meta-regression will be used for exploring the sources of heterogeneity. If there is no clinical heterogeneity, the Mantel–Haenszel random effects model will be used to perform meta-analysis.^[21]^ We will examine reporting bias using the Begg's and Egger's funnel plot method.^[23,24]^ Additionally, we plan to use the contour-enhanced funnel plot as an aid to distinguish asymmetry, if some more other factors leading to publication bias.^[25]^

### 3.10 Network meta-analysis

First, in order to ensure that an NMA is feasible, we will draw a network plot to describe and present the geometry of the treatment network of comparisons across trials. Trials will be excluded if the trials are not connected by treatments. In the network geometry, nodes represent different interventions, and edges represent the head-to-head comparisons between interventions. The size of nodes and thickness of edges are associated with sample sizes of intervention and numbers of included trials, respectively.

Next, the NMA will be conducted based on a Bayesian framework using the code invented by Dias et al^[26]^ through WinBUGS 1.4.3 software (MRC Biostatistics Unit, Cambridge, UK) to combine the direct evidence within trails and the indirect evidence across trails, as well as rank the efficacy of all feasible NPATs. The pooled estimation and the probability of which treatment is the best will be obtained using the Markov Chain Monte Carlo method. Three Markov Chains will be run simultaneously with different arbitrarily chosen initial values. We will first generate 50,000 simulations for each chain, and these simulations will then be discarded as the ‘burn-in’ period. Then posterior summaries will be based on 100,000 subsequent simulations. The model convergence will be assessed by trace plots and Brooks-Gelman-Rubin plots.^[27]^ The statistical heterogeneity in the entire network will be assessed on the bias of the magnitude of heterogeneity variance parameter (*I*^2^ or τ^2^) estimated from the NMA models using R-3.2.2 software (R Foundation for Statistical Computing, Vienna, Austria). The results of dichotomous outcomes will be reported as posterior medians of OR with 95% credible intervals (CrIs), and medians of MD with 95% CrI for continue outcomes. If a loop connecting 3 arms exists, inconsistency between direct and indirect comparisons will be evaluated using a node splitting method.^[28]^ The choices between fixed and random effect models, consistent and inconsistent models, will be made by comparing the deviance information criteria (DIC) for each model.^[26,29]^ The model with the lowest DIC will be preferred (differences >3 are considered significant).

Clinical decisions about the choices of treatments can be recommended based on the probability results of ranking when the differences in effect size of different treatments are small.^[30]^ The surface under the cumulative ranking area (SUCRA) will be calculated to summarize and report the probability values. SUCRA values are expressed as percentages—SUCRA value will be 100% for the best treatment, while SUCRA value will be 0% for the worst treatment.^[31]^

In order to explore the sources of heterogeneity or inconsistency in the entire network, we will perform network meta-regression or subgroup analysis. Network meta-regression will be conducted using random effects network meta-regression models to examine potential effect moderators such as follow-up and sample size.

If we include enough trials per comparison, a sensitivity analysis will be conducted. We will conduct a sensitivity analysis excluding trials that are missing relative data, and we will conduct another sensitivity analysis excluding trials with a total sample size of <50 randomized patients.

### 3.11 Grading of quality of evidence

The GRADE (Grading of Recommendation, Assessment, Development and Evaluation) approach will be used to assess the quality of evidence which presents the confidence we have about the effect estimation.^[32]^ The process will be performed on the platform of GRADEpro—GDT (https://gradepro.org/).

## 4 Ethics and dissemination

### 4.1 Ethical issues

Ethical approval and patient consent are not required since this is a meta-analysis based on published studies. We will submit our NMA to a peer-reviewed journal for publication.

### 4.2 Strengths and limitations of this study

- As far as we know, this is the first NMA which compares the efficacy of different NPATs for enhancing QoL of patients with breast cancer receiving chemo- and/or radio-therapy.
- The results of this NMA will assist clinicians and patients to make the best choice of NPATs for the patients with breast cancer.
- Our conclusion will rely on both the quality and quantity of the original studies available for review.

## Author contributions

Contributors: Conception and design of this systematic review and Bayesian network meta-analysis (Zhiyun He, Ailin Song, and Zhongtao Zhang); tested the feasibility of the study (Zhiyun He, Ailin Song, Xi Lv, and Yumin Li); developed the search strategy (Youcheng Zhang, Xiaokang Liu, Lei Zhao, Guosheng Ren); drafted this protocol (Zhiyun He, Ailin Song). All authors provided critical revisions of the protocol and approved the final manuscript.

**Conceptualization:** Zhiyun He, Ailin Song, Zhongtao Zhang.

**Methodology:** Zhiyun He, Youcheng Zhang, Xiaokang Liu, Lei Zhao, Guosheng Ren.

**Supervision:** Yumin Li.

**Validation:** Ailin Song, Xi Lv.

**Writing – original draft:** Zhiyun He, Ailin Song.
